# Violence against women in North-East Piedmont, Italy: a cross-sectional study on patients accessing the emergency department (2017–2020)

**DOI:** 10.1007/s00414-025-03644-6

**Published:** 2025-11-12

**Authors:** Elena Rubini, Giulia Facci, Stefano Cenati, Edit Shahi, Antonella Tedesco, Claudio Didino, Liliana Maglitto, Sarah Gino

**Affiliations:** 1 CRIMEDIM - Center for Research and Training in Disaster Medicine, Humanitarian Aid and Global Health, Università del Piemonte Orientale, 28100 Novara, Italy; 2 Department for Sustainable Development and Ecological Transition, Università del Piemonte Orientale, 13100 Vercelli, Italy; 3 Department of Health Sciences, Università del Piemonte Orientale, Novara, 28100 Italy; 4 Emergency Department, Azienda Socio-Sanitaria Territoriale della Valle Olona, 21052 Busto Arsizio, Varese, Italy; 5 Direzione Medica dei Presidi Ospedalieri, AOU Maggiore della Carità, 28100 Novara, Italy; 6 Responsabile SOS Direzione Sanitaria Presidio ospedaliero di Verbania, 28922 Verbania, Italy; 7 Medicina e Chirurgia d’Accettazione e Urgenza, Presidio Ospedaliero di Borgomanero, Borgomanero, 28021 Italy; 8 Direzione delle Professioni Sanitarie, ASL Verbano Cusio Ossola, 28845 Domodossola, Italy

**Keywords:** Gender-based violence, Violence against women, Emergency department, Triage, Clinical forensic

## Abstract

**Introduction:**

Violence against women (VaW) is a form of gender-based violence (GBV), a violation of human rights, and a public and global health issue. VaW leaves short and long-term sequelae on the health of survivors and necessitates correct clinical and forensic management. Victims of GBV can access care through the emergency department (ED), which is often the setting where disclosure of abuses occurs. This study aimed at documenting the epidemiology of patients accessing the ED for cases of VaW, as well as to understand the best practices and issues encountered in different health facilities in North-East Piedmont, Italy. This could contribute to documenting local data on the characteristics of survivors of VaW accessing emergency care, as well as to understanding the challenges encountered by healthcare staff in managing and documenting these cases at the ED level.

**Methods:**

A retrospective, cross sectional study covering the years 2017–2020 in different hospitals in North-East Piedmont (Novara, Borgomanero, Biella, Verbania-Cusio-Ossola) was conducted. Data on adult female patients (age > 18) was extracted in anonymized form from health facilities’ databases and analyzed using Stata18. Descriptive statistics summarized the characteristics of the pool of users and access to the ED.

**Results and conclusions:**

Patients accessing care in different EDs in North-East Piedmont described in many cases episodes of physical or physical and psychological violence perpetrated by partners in their homes. Many cases of minors witnessing violence were reported. Data from the years 2017–2020 showed that improvements were still needed in the management of GBV survivors in the ED, especially for what concerned assignment of triage color codes, proper documentation of the dynamics of violence, of the physical lesions, and other health sequelae.

*****What is already known***:**

- Victims of gender-based violence may access care through the Emergency Department.
- Healthcare providers in Emergency Departments are often the first professionals to whom victims disclose violence. They should be trained to identify victims of gender-based violence following existing guidelines and referrals to specialists should be available.
- Violence against women is a recognized public health issue that can lead to serious clinical and social consequences for women.

*****What this paper adds***:**

- This paper describes the characteristics of patients accessing emergency care for cases of violence against women in Northeast Piedmont.
  This paper demonstrates that the triage, documentation of injuries, and referral practices regarding victims of gender-based violence in the Emergency Department need improvement.
- This paper shows the issues connected with documentation of the sequelae of gender-based violence in Emergency Departments.

**Supplementary information:**

The online version contains supplementary material available at 10.1007/s00414-025-03644-6.

## Introduction

Gender-based violence (GBV) refers to any type of harm that is perpetrated against a person or group of people because of their factual or perceived sex, gender, sexual orientation, and/or gender identity [1, 2]. Violence against women (VaW) is defined as any act of GBV targeting women [2, 3]. Other intersectional^1^ identities or characteristics exacerbate the risk for some sub-groups to be subjected to GBV and for their specific needs to be overlooked (e.g., transgender populations, migrants, people with low socioeconomic status, people living with disabilities, elderly people, people with sexual orientation different from heterosexual) [4–8]. Particular contexts such as conflict, migration, and disasters can also increase the possibility to encounter GBV [9–14].

Intimate partner violence (IPV) is the most frequent type of VaW perpetrated by current or former partners [15] and is estimated to occur in one in three women (33%) [16], though it can also affect other populations (e.g., one in five men, 20%) [17, 18]. Domestic violence refers to all acts of physical, sexual, psychological, or economic violence that occur within the family or domestic unit regardless of relationship or cohabitation status between victim and perpetrator [19]. Both men and women are described in literature as perpetrators of abuse, even though the former are the most prevalent group [17, 20].

GBV and VaW are human rights violations and include among others physical, psychological, sexual, and economic violence as well as harmful traditional practices [2, 21]. They can result in immediate and long-term negative outcomes on the physical, mental, and social dimensions of the health of survivors [22]. Apart from different kinds of acute injuries, long-term sequelae can include chronic health problems, mental distress, as well as challenges in negotiating safer sex, and can escalate to fatal outcomes in the case of partner homicides, including femicides [22–28]. With regards to mental health consequences of GBV, they can include posttraumatic stress disorder, substance abuse, and suicide [24, 29]. GBV constitutes a public and global health issue [30, 31]. Namely, survivors tend to access curative care more often compared to the general population and seek preventive care less often, potentially hindering their health [22, 32–35].

Survivors of GBV can seek care through general (e.g., Emergency Departments, EDs) or specialist services [1, 19]. The former tend to prioritize the clinical management of patients, while the latter provide coordinated health and judiciary service provision through high quality forensic examinations [1, 19, 36, 37]. In Europe and in Italy the limited number and geographical spread of specialist services available cause most survivors to access care in EDs [13, 37, 38]. Disclosures of abuse often occur in this setting, even though fear of their consequences (e.g. concerns that ED staff will inform the police or that perpetrators will learn about the consultation) or of societal expectations (e.g., leaving an abusive partner, reporting to the authorities) may prevent survivors from doing so [30, 39]. Health providers in EDs have a pivotal role in the management of GBV cases and in creating a safe environment for survivors [40, 41]. Since negative personal beliefs and attitudes towards patients victims of GBV hinder quality of care and given the numerous challenges in their management (e.g., staff confidence, competence, time to allocate for the procedures, and physical resources such as adequate rooms for the assessment, and forensic documentation tools), education and training activities targeting health professionals have been implemented over the last decade [42–47].

The Istanbul Convention aimed at preventing, prosecuting, and eliminating VaW and domestic violence, discrimination, as well as at providing protection and assistance to victims [19]. Article 11 urged States to collect disaggregated data on GBV [19]. Italy ratified the Istanbul Convention in 2013 (Law n. 77, June 27, 2013) [48]. In 2019, the Ministry of Health and the Italian National Institute of Statistics (ISTAT) established a collaboration in order to document and monitor data related to GBV, including VaW, at national level, based on ED accesses and collected through the “*flusso informativo dell’Emergenza Urgenza”* (EMUR) [38, 49]. Italian law n. 53/2022 mandates public health facilities, and in particular EDs, to collect data on VaW [50]. Data collected by ISTAT is not disaggregated at regional nor at territorial level, and for this reason it is difficult to understand the specificities of each territory. North-East Piedmont, an area in the Piedmont region (North-West Italy), is no exception, and no study has been yet published on data from this geographical area. Moreover, studies conducted in this region analyzing data on GBV cases tend to be conducted in larger hospitals in the capital, Turin [13, 51–53], while smaller facilities, such as those present in North-East Piedmont, are usually overlooked.

The study aims to understand how EDs manage victims of VaW. The characteristics of victims of VaW accessing care in the ED of six different hospitals in North-East Piedmont and the best practices and issues in their management will be described.

The study was guided by two questions, namely “What are the characteristics of patients accessing care in the ED in North-East Piedmont after episodes of VaW?” and “What challenges emerged in managing victims of VaW in EDs in North-East Piedmont?”. Giving an answer to these questions will contribute to documenting local data on the epidemiology of survivors of VaW accessing care, as well as to understanding the issues connected to managing and documenting these cases in the ED.

## Methods

### Study design

This study is a retrospective, cross sectional study of data from six different EDs in North-East Piedmont, North-West Italy, covering the period 2017–2020.

### Study setting

#### Italy

In recent years, several normative changes have been made in Italy for the management of GBV and VaW cases, including in the healthcare sector. These encompass the “Red Code” (e.g. *Codice rosso*) Law 69/2019 that introduced new offenses (e.g., illegal dissemination of sexually explicit images or videos, disfigurement as a result of permanent injuries, coercion or inducement to enter marriage, and breach of the injunction to stay away from the family home and of the prohibition to visit places frequented by the offended person), mandated police training regarding GBV, and strengthened protective measures for victims in the Penal Code and the Criminal Procedure Code [54, 55].

The “National guidelines on socio-sanitary assistance to women victims of violence” (DPCM 24/01/2018) established the mandatory attribution of at least a yellow/orange code or equivalent (e.g., urgent care needed) during triage for patients presenting in EDs for cases of GBV in order to ensure charge taking in no longer than 20 min (which have now been reduced to 15 min), to avoid them voluntarily leaving the ED [56]. Law 208/2015 implemented the creation of a “Pink path” (e.g., *Percorso rosa*), with a protected access to ED through the attribution of a “Pink code” (e.g., *Codice rosa*) for those disclosing episodes of violence [57]. Moreover, a risk evaluation is conducted through the Brief Risk Assessment for the Emergency Department, a five item questionnaire and, depending on the patient’s situation and needs, operators can refer victims to shelters or in a brief intensive observation area in the ED [58]. Educational and training activities for health operators working with survivors of GBV have been implemented [46, 58].

#### Piedmont, North-West Italy

A regional network for the prevention and management of victims of sexual and domestic violence (*Coordinamento della rete sanitaria per l’accoglienza e presa in carico delle vittime di violenza sessuale e domestica della Regione Piemonte*) was implemented in 2009 in Piedmont [59]. Among the initiatives, since 2018 the regional network coordinated a series of awareness raising educational and training activities on GBV for health professionals, including an advanced course for those among them who more frequently manage cases of GBV. The network also developed regional standards for the management of patients who endured episodes of GBV [60].

From a legislative perspective, following two strategic national plans on VaW [61, 62], Piedmont established two plans against GBV covering a period of three years (2017–2019 and 2022–2024 respectively) [60, 63]. Among their objectives, the plans aim at promoting educational and training activities for health and social workers, in particular those operating in EDs.

After the approval of a law on preventing and contrasting GBV (Regional Law n. 4, February 24th, 2016), specific pathways for the management of victims of violence were implemented, together with the creation of multidisciplinary groups aiming at supporting their needs from a legal and socio-sanitary perspective. The law also introduced the possibility for survivors to receive psychological support in order to support them escaping a situation of violence [64].

#### The present study

**Fig. 1:**
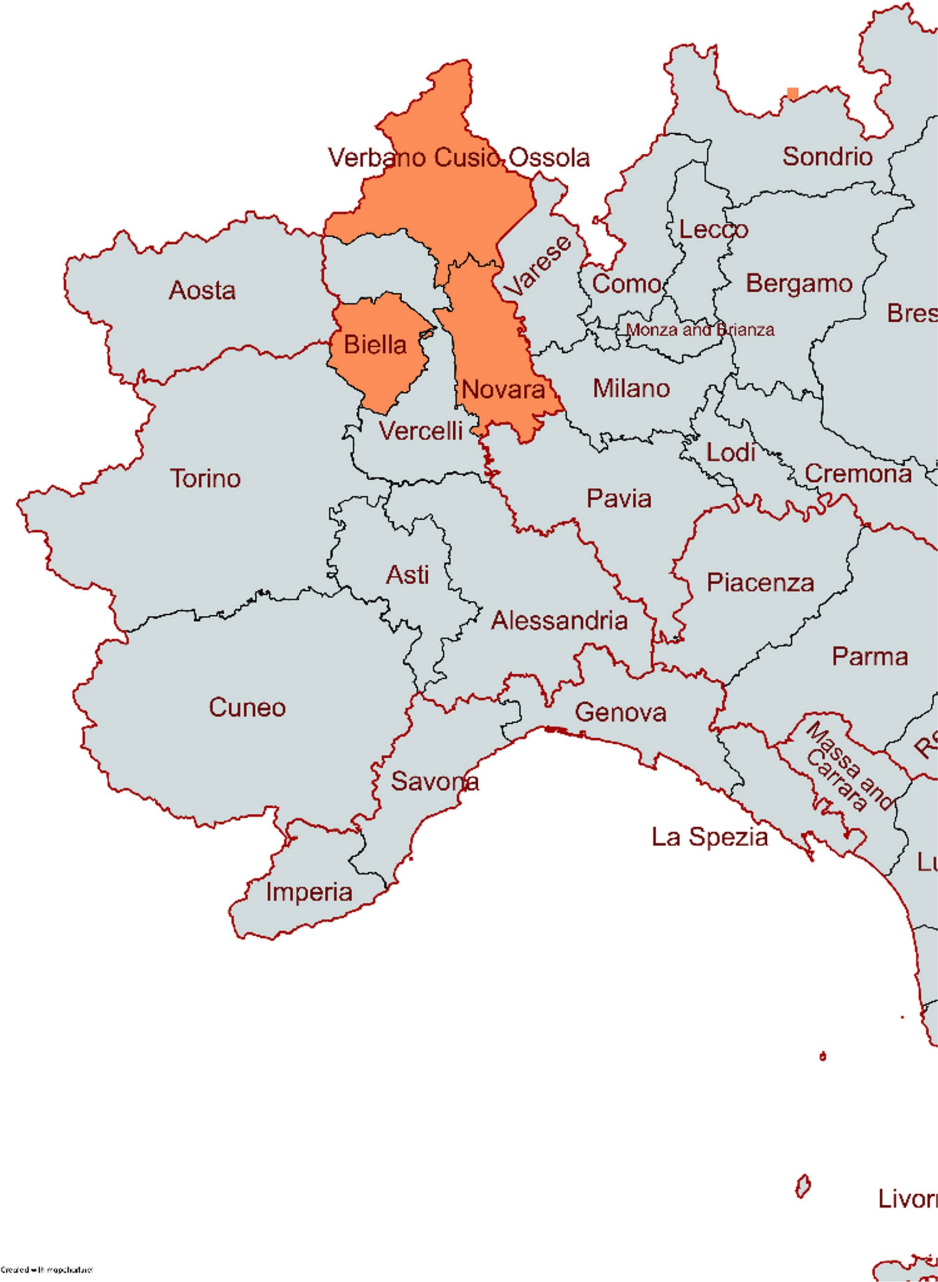
Map showing the location of the different provinces where the hospitals included are situated

Data was collected from the databases of the ED of six hospitals situated in three different provinces (e.g., second-level administrative divisions within regions), namely Novara, Biella, and Verbano-Cusio-Ossola (Fig. 1). Information on the name of the facility, their location, the province, and the acronym used in the study to refer to the facility can be found in Table 1. For VCO, the data for the three hospitals in Verbania-Pallanza, Domodossola, and Omegna were analyzed.

**Table 1:** Information on the facility, its location, province, and acronym used for reference in the study

Healthcare facility	Location	Province	Reference in the study
Azienda Ospedaliera Universitaria Maggiore della Carità	Novara	Novara	NO
Ospedale Santissima Trinità	Borgomanero	Novara	BRG
Ospedale degli Infermi	Ponderano (Biella)	Biella	BI
Ospedale Giuseppe Castelli	Verbania-Pallanza	Verbano-Cusio-Ossola	VCO
Ospedale San Biagio	Domodossola	Verbano-Cusio-Ossola	VCO
Ospedale Madonna del Popolo	Omegna	Verbano-Cusio-Ossola	VCO

### Data collection, screening, and eligibility criteria

Data referring to adult female patients (age > 18) accessing care for cases of GBV between 2017 and 2020 was collected from the ED databases of each hospital, extracted in anonymized form in a spreadsheet (Supplementary material 1). Data from the three hospitals in the Verbano-Cusio-Ossola province was reported in aggregated form.

Entries related to patients under the age of 18 at the time of access to the ED were excluded, as the management of these cases in Italy is regulated differently from a clinical and forensic perspective.

### Statistical analysis

Descriptive statistics, including frequencies, proportions, and measures of central tendency (e.g., mean), were used to summarize the characteristics of the victims, the events, the aggressors, and the ED access details. Specifically, characteristics of the victims, including age, nationality, and pregnancy status, were analyzed to provide an overview of their demographic profiles. Situational aspects of the episodes, such as the type of violence, location, presence of witnesses, and whether the episode was recurrent, were also summarized to better understand the contexts in which violence occurred. Information on aggressors, including age and relationships to the victim, was evaluated. ED access characteristics were also examined, including mode of arrival (ambulance, police, or self-presentation), triage code, prognosis, types and locations of physical injuries, presence of psychological symptoms, discharge diagnosis and type of service delivered (e.g. referral, imaging testing, specialist examination, injury management).

To preserve the integrity of the data, missing values were neither imputed nor excluded, always allowing for a transparent presentation of the available information. During data cleaning, free-text fields were recoded into categorical variables. Categories with a count of zero indicate that no entries matched that classification after the recording. The expression “n.a.” was used in the table for variables that were not collected by the included centers.

As some variability existed in data collection practices across hospitals, the descriptive approach was suitable for providing a comprehensive overview of the characteristics and contexts of cases within the dataset.

### Operational definitions

In the present study, the terms “survivor” and “victim” will be used interchangeably to refer to individuals subjected to GBV; the first term serves to highlight the process of recovery, while the second calls attention to the criminal nature and the severity of acts of GBV [10].

The expressions “non-Italians”, “migrants”, and “individuals of foreign origin” will be used to refer to victims of nationalities other than Italian.

In the study, triage color codes referred to will be “green” for nonurgent treatment needed, “yellow” for urgent treatment needed, and “red” for immediate threat to life [65].

### Ethics

The study was approved by the Novara Intercompany Ethics Committee [*CE 116/19 & CE 28/20*] and complies with the Declaration of Helsinki for experiments involving humans (2013), to the General Data Protection Regulation (2018), and to the Provision no. 146/2019 of the Italian Privacy Guarantor. For information about consent to participate, please see the Informed Consent Statement.

## Results

Supplementary material 2 reports data on the characteristics of the victims, of the episode of GBV, aggressors, and access to the Emergency Departments of Verbania-Cusio-Ossola (VCO), Novara (NO), Borgomanero (BRG) and Biella (BI) during the years 2017–2020. In the following sections, the most relevant findings related to characteristics of the abuse, victims, and perpetrators as well as those connected to the access to the ED will be illustrated by presenting data in weighted averages.

### Characteristics of the abuse, victims, and perpetrators

The more reported forms of GBV were physical violence and physical violence in association with psychological abuse (45% each). The most described weapons were body parts (84%), followed by an association of them with other blunt force objects (5%), and blunt force objects alone (3%).

There was a limited number of women accessing care while pregnant (3%). Among the cases documented in our dataset, migrant women were, on average, 6.5 years younger than Italian women. The more documented group of non-Italian patients accessing care was Moroccan nationals (26%). The other two groups frequently accessing the ED for GBV cases originated from Sub Saharan Africa and Eastern Europe.

Concerning the nationality of aggressors as described by patients, in the three databases reporting this data they were in most cases Italians. Many victims described their partners (52%) as well as former partners (15%) as perpetrators of violence. Non-Italians reported less frequently being attacked by former partners compared to Italian victims and disclosed conversely being more victimized by partners compared to their Italian counterparts. These data on the relationship between victim and aggressor being mostly in the context of a current or former intimate relationship were also confirmed by another element, namely the place where violence occurred, which, when reported in the database, was in most cases the survivors’ home.

While in other health facilities the terms used in Italian by patients to describe perpetrators identified them as males, in one database (VCO) they were in 6% of cases females. Another interesting element was that overall, in 11% of cases the aggressor was generically described as a “known person”, an expression that does not convey their relationship with the victim.

Alarmingly, minors constituted more than half of the overall witnesses (54%). However, in the data presented it was not possible to understand whether children also accessed care at the ED on the same occasion after enduring witnessed violence.

For what concerns past victimization, available figures varied (51% on average), reaching 61–89% in two EDs. Only in Borgomanero it was specified that in 92% of cases victims were attacked by the same perpetrator. It is not possible to understand the reason why in the other two EDs figures for revictimization are significantly lower (6%−17%).

### Access to the ED

As for what concerns access to the ED, most patients arrived via private transport (68%), followed by ambulances (22–33%). Only in one of the hospitals, interestingly the one where the longer prognoses were attributed, did a greater number of patients arrive accompanied by the police compared to the other facilities (18%, Biella).

Most patients received a green code (73%) at triage, followed by a yellow or equivalent code (25%).

Taking into consideration disaggregated data for the different hospitals, a yellow or equivalent code was assigned only in Novara in 71% of cases, while the other two had extremely low rates of yellow or equivalent codes (6% in VCO and 2% in Borgomanero).

In two hospitals (3% vs. 7% in VCO, 24% vs. 32% in Novara), foreign nationals had lower rates of attribution of a yellow code compared to Italians.

Mean length of prognosis was short (8.61 days on average), and even when it was ≥ 20 days there were high rates of green codes during triage (80% on average).

Documented physical injuries were preponderantly at dermatological and soft tissue level (24–88%) followed by musculoskeletal lesions and algias (8–25%). Nevertheless, diagnostic imaging testing (e.g., X-ray, CT scan, ecography) was the examination most requested by the ED physicians (36–70%) followed by specialist examinations and lab tests. The higher number of diagnostic imaging testing compared to that of the musculoskeletal lesions is probably due to suspicious cases where diagnostic imaging testing was requested but turned out negative after the examination.

In most cases lesions involved multiple regions of the body, while single ones targeted mostly upper and lower limbs and extremities and head and neck. Overall, the dimensions and characteristics of the lesions were rarely documented in detail (2%) and photographed (7%).

Documentation of psychological symptoms and signs occurred only in 42% of cases. As for what concerns referral to psychological or social support services, they were never mentioned in the databases.

When reported, the most mentioned discharge diagnoses, as thematically categorized by the research team, varied considerably among the different facilities, being "symptoms and signs" in VCO, "violence or aggression" in Novara, and "GBV" in Borgomanero. Importantly, in VCO and Novara GBV was rarely mentioned in the discharge diagnosis (4–9%) both directly or describing in detail the episode of violence. From the available data it was not possible to holistically understand whether a safe discharge plan was established.

## Discussion

In our study, physical violence and physical violence in association with psychological abuse were the most reported, aligning with the current literature. A systematic review of prevalence studies on GBV found EDs to be the setting where survivors disclosed more past episodes of physical violence, second only to psychiatric wards and obstetrics and gynecology clinics [66]. The same study revealed the rates of disclosure of lifetime emotional violence to be the highest in EDs [66].

The most described weapons were different forms of blunt force objects. This is in adherence to the literature documenting blunt force as the more frequent cause of acute injury patterns among interpersonal violence victims, including in GBV cases [67–71].

In our dataset, a limited number of women accessed care for GBV while pregnant, despite this condition potentially exacerbating the risk for GBV and even though a pregnant woman has more probability to suffer from GBV than from gestational diabetes or pre-eclampsia [53, 72–75]. This may be related to them accessing the obstetric and gynecological ED directly and their access not being registered in the databases analyzed in the study. Other provider-related factors leading to an underestimation of cases of GBV during pregnancy could include limited educational and training initiatives targeting healthcare professionals on this issue [53].

Aggressors were described as Italians in most cases in the three databases reporting their alleged nationality. This is in accordance with data presented in a report by the Italian Ministry of the Interior concerning the years 2022–2023, where perpetrators of physical and sexual violence and stalking were identified as Italians in 72% of cases [76].

Partners and former partners were reportedly perpetrators of violence, in adherence with the current literature, with IPV being the most prevalent form of GBV worldwide [15, 16, 77, 78]. The fact that non-Italians reported less frequently being attacked by former partners and being more victimized by partners compared to their Italian counterparts could be related to their different cultural background, in particular to beliefs and norms around the possibility women have of choosing to leave an abusive relationship, as well as acceptance of violent behaviors [79–81], or to characteristics related to other intersectional identities, for example connected to their legal status (e.g., their permit of stay depending on their marital status and on the visa of their husbands) [82–86].

In one database almost 25% of aggressors were females. Even though it is well-reported in the literature that most abusers in cases of GBV are men, studies have emerged documenting the presence of women aggressors targeting both men and women [17, 20, 87–90].

The use of the expression “known person” - which does not convey the relationship between aggressor and victim - could be related to reticence on the part of the survivors or conversely being connected to healthcare workers preferring to avoid the medico-legal component of the management of GBV and focusing only on health-related elements.

Revictimization rates were on average high in our population. Other studies have analyzed the issue of revictimization by focusing on the characteristics of aggressors and victims. Abusers’ recidivism rates in cases of IPV are high, reaching 15–60% [91]. Literature on revictimization in cases of GBV is scarce, especially when focusing on vulnerability factors for multiple partner victimization [92, 93]. However, the chronicity of IPV has been recognized in association with some intersectional risk factors (e.g., socioeconomic status, level of education, unemployment) [94–96]. Variation in revictimization rates among EDs may depend on the ability of healthcare workers to document past victimization, from patients avoiding disclosing past episodes of abuse, or from actual smaller revictimization rates [92].

Most patients received a green code at triage, followed by a yellow or equivalent code, in alignment with the data shown in the report on Violence and access to the ED in the years 2017–2019 promoted by the Ministry of Health and the Italian Institute of Statistics [38].

Given that part of our data was collected before the enactment of “National guidelines on socio-sanitary assistance to women victims of violence” (DPCM 24/01/2018), which established a mandatory attribution of a yellow or equivalent code to patients disclosing episodes of GBV, figures with low attribution of a yellow or equivalent code in two of the hospitals and the attribution of a green code in 29% of cases in another facility highlight for how long this aspect has been underestimated, despite the number of short and long term health consequences provoked by GBV [56]. On the other hand, the attribution of a green code could be related to sequelae being minor lesions and to patients not disclosing the event that caused the injuries at triage.

Lower rates of attribution of a yellow code in foreign nationals compared to Italians in some health facilities could possibly be related to an under triage, a common issue in migrants accessing the ED [97].

Dermatological and soft tissue level injuries and musculoskeletal lesions and algias were the most reported physical injuries. This is in adherence with the literature describing contusions, abrasions and lacerations among the most recurrent patterns of injuries in patients accessing the ED for IPV cases, together with musculoskeletal ones [98, 99].

Single lesions were situated in the upper and lower limbs and extremities as well as head and neck. Head, neck, and facial injuries are recognized in the literature among the patterns of lesions specific for IPV [100–103] and are among the most documented physical sequelae in cases of IPV together with injury to the limbs and extremities, these last ones probably caused in many cases by an attempt of the victims to defend themselves [104, 100]. Head and limbs are also the anatomical regions most affected in cases of femicides [91, 101, 102].

In our study, health staff rarely documented the dimensions and characteristics of the lesions and photographed them. This is in accordance with the literature highlighting the difficulties that ED personnel have in managing the medico-legal elements in GBV and other types of interpersonal violence cases [36]. This reflection also applies to the other information documented in the databases, which varied considerably among health facilities.

Referral to psychological or social support services were never mentioned in the databases, highlighting an underestimation of psychological and social components of health by clinical staff or to the difficulties connected with referral paths [103].

### Limitations

This study has some limitations that should be considered. First, data presented does not fully capture the extent of VaW in North-East Piedmont, as it is limited to survivors who access care in EDs. More studies encompassing data at territorial level could provide a more accurate description of the phenomenon. Second, since data on the gender of patients was not reported in the databases, the analysis does not encompass this variable. For this reason, we referred to “female patients” focusing on the sex they were assigned at birth, limiting our research to cisgender women. The collection of data disaggregated per gender would allow for an analysis of GBV affecting also other groups (e.g., transgender women and men). Third, the data were occasionally fragmented and inconsistently collected both within the same hospital and across facilities, which may have impacted the reliability and completeness of the findings. In particular, there were missing values for key variables such as type of violence and use of weapons, limiting the depth and robustness of some analyses. Additionally, many variables were originally recorded as open-text entries, requiring recategorization by the research team. This process may have introduced classification bias and reduced the consistency and comparability of the data. This variability highlights the need for standardized data collection practices in the healthcare setting, particularly for episodes of GBV and aggression. Fourthly, the use of a quantitative methodology with the limited data available in ED databases prevents us from properly understanding the conditions of patients at the time of accessing care.

### Recommendations

The EDs can play a key role in identifying and supporting victims of GBV, and could act as entry points for public health interventions aimed at informing, protecting, and empowering women. However, to effectively do so, both operational and research efforts are needed. First, ED staff should receive appropriate training to recognize signs of violence, including non-normative patterns of aggression and victimization, and to collect relevant data in a systematic and trauma-informed manner. This requires investments in ongoing education, and the integration of standardized clinical pathways that reduce reliance on subjective elements. Second, standardized protocols supporting data collection, clinical decision-making, and referrals should be implemented. These should be supported by digital tools that reduce the administrative burden on healthcare staff, already operating under significant time and resource constraints. Third, in cases where violence is either suspected or disclosed, ED staff should be able to provide appropriate information. Awareness-raising and educational resources should be made available in different languages to survivors accessing the ED, explaining what types of support are available for leaving a situation of violence. Given the high proportion of revictimization, the implementation of strategies that prevent reiteration of violence and dependence from the abuser is essential. This requires a strong collaboration between health, social, and legal services.

Finally, further research is needed on underexplored populations or topics, including migrant women, male victims of IPV and domestic violence, as well as violence from female perpetrators. Other studies should be conducted to document the characteristics of pregnant women experiencing GBV, as well as analyzing in parallel the access to the ED of mothers who are victims of GBV and that of their children. Future studies should analyze whether the assignment of the yellow/orange code increased during the years 2021–2024.

## Conclusion

This cross-sectional study described the characteristics of victims, aggressors, episodes of GBV and access to care through different EDs in North-East Piedmont in the years 2017–2020. While Piedmont implemented policies and operational methodologies aiming at ensuring a high standard charge-taking of patients who endured GBV, the findings for the years 2017–2020 still show room for improvement, in particular for what concerns the proper documentation of the physical lesions and psychological health sequelae, as well as of the dynamics of violence — both during triage and in the discharge diagnosis — and the relationship between victim and perpetrator. At triage, many patients were assigned a green code, possibly hindering quick access to care. Other studies should analyze whether in the years 2021–2025 the situation has improved, in terms of the correct attribution of the color code at triage and following the educational and training initiatives implemented, including for what concerns the documentation of the lesions through photographs.

## Supplementary Information

Below is the link to the electronic supplementary material.

Supplementary Material 1 (DOCX 48.2 KB)

Supplementary Material 2 (DOCX 35.6 KB)

## Data Availability

The datasets used and/or analyzed during the current study are available from the corresponding author on reasonable request.

## Acronyms

BI: Biella
BRG: Borgomanero
CE: comitato etico
ED: emergency department
EMUR: informative system for monitoring and assistance in emergency and urgent care
DPCM: Decreto del Presidente del Consiglio dei Ministri
GBV: gender-based violence
IPV: intimate partner violence
ISTAT: Italian National Institute of Statistics
NO: Novara
VaW: violence against women
VCO: Verbano-Cusio-Ossola

## Footnotes

1 Intersectionality refers to a framework conceptualized by Kimberlé Crenshaw, recognizing that people’s lives are shaped by their identities, relationships and social factors. These combine to create intersecting forms of privilege and oppression depending on a person’s context and existing power structures such as patriarchy, ableism, colonialism, imperialism, homophobia and racism. It also refers to the interaction and cumulative effect of multiple forms of discrimination.
