# Supplementary material for: Violence against women in North-East Piedmont, Italy: a cross-sectional study on patients accessing the emergency department (2017–2020)
Source: Int J Legal Med. 2025 Nov 12;140(2):1153–65. doi: 10.1007/s00414-025-03644-6 (PMC12957630; doi:10.1007/s00414-025-03644-6)
Supplement: Supplementary file 1 — Supplementary Material 1 (DOCX 48.2 KB) [file 414_2025_3644_MOESM1_ESM.docx]

Additional file 1. Data extracted from the hospital databases.

| **General information** |
| --- |
| Date of access  Kind of event  Location where the event occurred  Did a similar event occur in the past?  Who brought the patient to the ED?  Witnesses |
| **Anamnesis** |
| Nationality/Country of origin  Sex  Age  Pregnancy status (reported) |
| **Perpetrator** |
| **Access to ED** |
| Triage evaluation  Discharge diagnosis  Triage code  Health services provided  Therapy |
| **Medical examination of lesions** |
| Kind  Location  Characteristics  Dimensions  Prognosis  Was a photograph of the lesions taken? |
| **Psychological signs and symptoms** |
| **Alcohol use** |
| Voluntary  Forced |
| **Drug use** |
| Voluntary  Forced |
