# Supplementary material for: Violence against women in North-East Piedmont, Italy: a cross-sectional study on patients accessing the emergency department (2017–2020)
Source: Int J Legal Med. 2025 Nov 12;140(2):1153–65. doi: 10.1007/s00414-025-03644-6 (PMC12957630; doi:10.1007/s00414-025-03644-6)
Supplement: Supplementary file 2 — Supplementary Material 2 (DOCX 35.6 KB) [file 414_2025_3644_MOESM2_ESM.docx]

**Additional file 2.** Characteristics of the victims, of the episode of GBV, aggressors, and access to the Emergency Departments of Verbania-Cusio-Ossola (VCO), Novara (NO), Borgomanero (BRG) and Biella (BI) during the years 2017-2020. Percentages are presented in rounded numbers.

|  | **VCO (N = 212)**  **n (%)** | **NO (N = 148)**  **n (%)** | **BRG (N = 196)**  **n (%)** | **BI (N = 216)**  **n (%)** |
| --- | --- | --- | --- | --- |
| **Victim’s demographics** | | | | |
| **Age (mean and SD)** | 41.9 years (SD = 14,1) | 40.4 years (SD = 13.4) | 40.4 years (SD = 10.8) | 39.4 years (SD = 12.3) |
| **Nationality** | | | | |
| Italian | 174 (82%) | 83 (56%) | 147 (75%) | 132 (61%) |
| Foreign | 38 (18%) | 65 (44%) | 49 (25%) | 68 (31%) |
| Missing | 0 (0%) | 0 (0%) | 0 (0%) | 16 (7%) |
| **Pregnancy status** | | | | |
| Pregnant | 6 (3%) | 3 (2%) | 1 (0.5%) | 12 (6%) |
| Missing | 0 (0%) | 0 (0%) | 79 (40%) | 14 (6%) |
| **Characteristics of the episode of violence** | | | | |
| **Type of violence** | | | | |
| Physical | 115 (53%) | 139 (94%) | 61 (31%) | 38 (17%) |
| Psychological | 26 (12%) | 0 (0%) | 7 (3%) | 8 (4%) |
| Physical and psychological | 61 (28%) | 5 (3%) | 123 (63%) | 160 (74%) |
| Sexual violence, sexual violence combined with physical and psychological | 4 (2%) | 4 (3%) | 5 (2%) | 10 (5%) |
| Missing | 10 (5%) | 0 (0%) | 0 (0%) | 0 (0%) |
| **Weapons** | | | | |
| Body parts | 185 (87%) | 131 (89%) | 158 (81%) | 174 (81%) |
| Blunt force objects | 11 (5%) | 7 (5%) | 4 (2%) | 3 (1%) |
| Cutting weapons | 0 (0%) | 0 (0%) | 2 (1%) | 0 (0%) |
| Body parts + blunt force objects | 8 (4%) | 1 (1%) | 11 (6%) | 16 (7%) |
| Blunt force objects + cutting weapons | 1 (0.5%) | 3 (2%) | 8 (4%) | 7 (3%) |
| Blunt force objects + fire weapons | 0 (0%) | 0 (0%) | 1 (0.5%) | 3 (1%) |
| Other (e.g., “thermic agents”, multiple weapons etc.) | 0 (0%) | 2 (1%) | 1 (0.5%) | 1 (0.5%) |
| No weapon | 0 (0%) | 0 (0%) | 3 (2%) | 0 (0%) |
| Missing | 7 (3%) | 4 (3%) | 8 (4%) | 12 (6%) |
| **Place where violence occurred** | | | | |
| Victim’s home | n.a. | 91 (61%) | 77 (39%) | 6 (3%) |
| Public outdoor place | n.a. | 18 (12%) | 11 (6%) | 2 (1%) |
| Workplace | n.a. | 7 (5%) | 0 (0%) | 0 (0%) |
| Other | n.a. | 7 (5%) | 16 (8%) | 3 (2%) |
| Missing | n.a. | 25 (17%) | 92 (47%) | 205 (95%) |
| **Witnesses** | | | | |
| Present | 43 (4%) | 14 (9%) | 112 (57%) | 108 (50%) |
| Of which minors (<18%) | 33 (77%) | 9 (64%) | 54 (27%) | 64 (30%) |
| Missing | 0 (0%) | 0 (0%) | 30 (15%) | 44 (20%) |
| **Prior episodes of GBV** | | | | |
| Yes | 64 (30%) | 25 (17%) | 174 (89%) | 132 (61%) |
| Of which from the same perpetrator | n.a. | n.a. | 92% | n.a. |
| Missing | 0 (0%) | 0 (0%) | 0 (0%) | 0 (0%) |
| **Perpetrator’s characteristics** | | | | |
| **Nationality** | | | | |
| Italian | 43 (20%) | n.a. | 50 (25%) | 112 (52%) |
| Foreign | 14 (7%) | n.a. | 22 (11%) | 29 (13%) |
| Missing | 155 (73%) | n.a. | 124 (63%) | 75 (35%) |
| **Gender** | | | | |
| Female | 13 (6%) | n.a. | n.a. | n.a. |
| Missing | 199 (94%) | n.a. | n.a. | n.a. |
| **Relationship with victims** | | | | |
| Partner | 92 (43%) | 45 (32%) | 137 (71%) | 126 (58%) |
| Former partner | 19 (9%) | 15 (11%) | 50 (26%) | 36 (17%) |
| Acquaintance | 32 (16%) | 16 (11%) | 3 (1%) | 4 (2%) |
| Family member | 25 (12%) | 7 (5%) | 2 (1%) | 5 (3%) |
| “Known person” | 26 (13%) | 45 (32%) | 0 (0%) | 10 (5%) |
| Stranger | 5 (2%) | 0 (0%) | 0 (0%) | 0 (0%) |
| Missing | 13 (6%) | 0 (0%) | 0 (0%) | 35 (16%) |
| **Characteristics of the access to the ED** | | | | |
| **Mean lenght of prognosis** | 8.1 days (SD = 6.8) | 7.9 days (SD = 6.8) | 7.3 days (SD = 5.2) | 11.2 days (SD 8.7) |
| **Psychological symptoms reported** | 3% | 7% | 86% | 63% |
| **Referral to psychological or social support services** | n.a. | n.a. | 24% | n.a. |
| **Type of physical lesions** | | | | |
| Dermatological and soft tissue bruises and injuries (e.g., contusions, abrasions, ecchymoses, bruises, etc.) | 187 (88%) | 147 (99%) | 113 (58%) | 51 (24%) |
| Musculoskeletal lesions and algias (e.g., traumatic injuries, fractures, joint impairments) | 21 (10%) | 1 (1%) | 49 (25%) | 17 (8%) |
| Other (e.g., general pain, hyperemia, epistaxis, etc) | 0 (%) | 0 (0%) | 6 (4%) | 5 (2%) |
| Missing | 4 (2%) | 0 (0%) | 28 (14%) | 143 (66%) |
| **Location of the physical lesions** | | | | |
| Limbs and extremities | 24 (11%) | 24 (16%) | 50 (25%) | 20 (9%) |
| Head and neck | 21 (10%) | 22 (15%) | 52 (26%) | 28 (13%) |
| Thorax | 1 (0.5%) | 2 (1%) | 4 (2%) | 0 (%) |
| Not specified/multiple lesions | 62 (29%) | 36 (24%) | 54 (27%) | 16 (7%) |
| Missing | 104 (49%) | 64 (43%) | 36 (18%) | 152 (70%) |
| **Examinations requested** | | | | |
| Imaging testing | 92 (43%) | 103 (70%) | 71 (36%) | n.a. |
| Laboratory tests | 27 (13%) | 11 (7%) | 11 (6%) | n.a. |
| Specialist examination | 41 (19%) | 9 (6%) | 62 (32%) | n.a. |
| Injury management (e.g., wound cleaning, wound dressing, stitching, etc) | 4 (2%) | 0 (0%) | 0 (0%) | n.a. |
| Missing | 48 (23%) | 25 (17%) | 52 (26%) | n.a. |
| **Discharge diagnosis** | | | | |
| GBV | 8 (4%) | 13 (9%) | 37 (19%) | n.a. |
| Physical violence or aggression | 47 (22%) | 97 (66%) | 16 (8%) | n.a. |
| Symptoms and signs (or an association of both) | 106 (50%) | 36 (24%) | 47 (24%) | n.a. |
| Other | 5 (2%) | 2 (1%) | 4 (2%) | n.a. |
| Missing | 46 (22%) | 0 (0%) | 92 (47%) | n.a. |
